# Light-Based Reaction Speed Does Not Predict Field-Based Reactive Agility in Soccer Players

**DOI:** 10.3390/jfmk10030239

**Published:** 2025-06-24

**Authors:** Adele Broodryk, Filip Skala, Retief Broodryk

**Affiliations:** 1 Physical Activity, Sport and Recreation Research Focus Area (PhASRec), Faculty of Health Sciences, North-West University, Potchefstroom 2520, South Africa; filip.skala@uniba.sk (F.S.); retief.broodryk@nwu.ac.za (R.B.); 2 Department of Biological and Medical Sciences, Faculty of Physical Education and Sport, Comenius University in Bratislava, 814 99 Bratislava, Slovakia

**Keywords:** reactive agility, sprint speed, sports vision, hand-eye coordination, soccer, perceptual–cognitive training, human vs. light-based reaction, ecological validity, decision-making

## Abstract

**Background:** The motor response to human visual stimuli is unique and differs from the reaction to light-based visual stimuli. While laboratory-based tests offer valuable insights into athletes’ basic perceptual–motor abilities, their translation to actual sports-specific tests is limited. Methods: Following a thorough warm-up, 44 collegiate-level male soccer players (age: 24.4 ± 2.5 y, mass: 63.01 ± 7.3 kg, stature: 167.62 ± 6.3 cm) from a tertiary institution completed the following tests: Sports Vision Test (20-light proactive speed test), 40 m sprint test (split times over 5, 10, 20 and 40 m), and a live Reactive Agility Test (RAT) entailing them to sprint, change direction either towards their dominant limb or non-dominant limb in response to a live tester, and sprint again. **Results**: Numerous moderate correlations were seen between the RAT and various sprint distances (r > 0.3, ES > 0.3, *p* < 0.05). The reaction speed relationship between the light-based (SVT) and live stimuli (RAT) test yielded a weak relationship (r > 0.4, ES > 0.5, *p* < 0.05). Furthermore, the light-based hand–eye coordination speed did not predict acceleration or top speed, while the total RAT time did explain 10.5% of top speed (40 m). No significant differences in the SVT average and total time were found among playing positions. **Conclusions**: The limited correlations observed indicate that light-based reaction training alone may not be sufficient to translate to field-based reactive agility; therefore, training should integrate perceptual–cognitive and motor demands. Future research should refine laboratory-based tests by incorporating contextual elements to enhance ecological validity and further investigate the transferability of these skills from controlled settings to real-world game scenarios.

## 1. Introduction

Soccer performance relies on physical abilities, such as explosive strength, linear speed, and change in direction (COD) [1,2], in combination with exceptional abilities in motor control, perception, and cognitive functioning [3]. Only 15–20% of high-intensity efforts are spent returning to the playing position. At the same time, the remaining majority are performed for tactical or technical purposes based on game circumstances [4]. Elite competitive matches consist of an average of 305 ± 50 CODs [5], performed in a changing environment, demanding athletes to react to external stimuli continuously. Therefore, athletes must rely on visual cues such as ball trajectory, teammate runs, and opponent positioning while operating under severe time pressure [6].

Recent studies have elucidated the importance of sensory and cognitive functions for soccer performance [3,7,8]. These aspects play a crucial role in agility performance, which in team sports differs from the pre-planned COD, i.e., planned agility [9]. Planned agility performance is dependent on linear speed, strength properties, and COD technique [10,11]. Reactive agility (RA), on the other hand, requires decision-making and perceptual processes [9]. RA can be evaluated using timing gates, either in combination with light sensors (e.g., Sports Vision Test—SVT) or human stimuli (such as live testers). Some studies prefer to use standardised light stimuli to trigger the COD movement [11,12] due to the potential bias of the human factor in the assessment procedure. Conversely, studies aiming to simulate the match environment prefer to use a live tester and argue that exposure to sport-specific cues and patterns enhances the ecological value of the reactive agility test (RAT) [13]. However, it seems that light-based and live RA tests measure different qualities [14,15].

More recently, RA performance measured through live stimuli differentiated between elite (ranked 5th and higher) and sub-elite (ranked 8th and lower) soccer players, whereas light-based RA only differentiated amateur players from national players [14]. Supported by the existing relationship between the level of cognitive functions and sport-specific motor skills [3], soccer performance might benefit from superior perceptual skills and fast reaction time in combination with players’ neuromuscular qualities. Given that soccer players must sprint explosively for one to three seconds, the significance of response time in player performance becomes even more apparent [16]. Unfortunately, simple, straightforward visual reaction tests are not a predictive parameter of speed performance [16], nor can they differentiate the performance levels of soccer players [17].

Adaptation to sport-specific training enables players to react faster in complex tasks, including choice reactions [18] and visual inhibition [19]. The interaction between sensory, cognitive, and motor functions plays a critical role in peripheral eye–hand coordination, as assessed through rapid responses to randomly generated light stimuli [20]. This evaluation offers insight into a player’s psychomotor functioning, highlighting their ability to integrate visual processing, decision-making, and motor execution in high-speed scenarios [20,21]. It is suggested that reaction time contributes to the reaction accuracy performance of soccer players when reacting to non-specific light stimuli in computer-based tasks [22]. Hence, the visual system interacts with cognition when soccer players are confronted with tasks that involve multiple reaction options [23], meaning that psychomotor performance may, to some extent, reflect the cognitive aspect of reaction time. Currently, several studies ascribe RA performance to the sensory and cognitive functioning of players [9,24]. However, evidence is lacking on whether athletes reacting fast to light-based stimuli correlates with sport-specific COD movements.

Therefore, this study aimed to examine the relationship between laboratory-based and field-based psychomotor reactive speed tests among soccer players, addressing the gap in studies comparing these tests for psychomotor functioning. The strength of the potential relationship could reveal whether lab-based (SVT) and field-based reactive agility tests (RAT) measure similar aspects of psychomotor functioning. The findings can inform future research on SVT and RAT applications while also assisting practitioners in applying these tools to assess reactive agility performance. Understanding this relationship holds implications for sports diagnostics, training methodologies, talent identification, and performance optimisation in sports.

## 2. Materials and Methods

### 2.1. Research Design

An analytical cross-sectional study design was employed to investigate the relationship between light-based visual stimuli and live-based visual stimuli, as well as speed over a specified distance. Participants first completed a thorough 15 min warm-up, which consisted of aerobic jogging, dynamic and ballistic stretches, and two repetitions of 30 m sprints at 80% of their maximal effort. After this, they completed a light-based visual test on a vision board. They then completed a speed test over 40 m, measuring the time splits at 5, 10, 20, and 40 m. This test was followed by a reactive agility test incorporating a live tester on both their left and right sides in a randomised order.

### 2.2. Participants

The study comprised 44 collegiate-level male soccer players (age: 24.4 ± 2.5 y, mass: 63.01 ± 7.3 kg, stature: 167.62 ± 6.3 cm, playing experience: 9.29 ± 1.2 years, defenders: *n* = 16, forwards: *n* = 14, midfielders: *n* = 10, goalkeepers: *n* = 4) from a tertiary institution in South Africa competing in a senior competitive amateur league. Ethical approval was obtained from the lead author’s institution’s ethical committee (NWU-00299-21-A1) before data collection commenced on 23 March 2023. Players reported no prior exposure to vision boards or the specific reactive agility test (RAT) employed in this study.

### 2.3. Physical Performance Tests

#### 2.3.1. Anthropometric Measurements

Body mass was recorded to the nearest 0.1 kg, using a calibrated BFW 300 Platform scale (Adam Equipment Co. Ltd., Milton Keynes, UK), with the subject wearing minimal clothing and no shoes. Body stature was recorded to the nearest 0.1 cm using a Harpenden portable stadiometer (Holtain Ltd., Crymych, UK) with the subject standing upright and the head in the Frankfort plane.

#### 2.3.2. The 40 m Speed Test

The 40 m sprint is a valid and reliable test (r = −0.73, r = 0.9, respectively) to measure maximal speed velocities as observed during a match [25]. Electronic timing gates (SmartSpeed, Fusion Sport, Milton, Australia) were set at 0, 5, 10, 20, and 40 m intervals to record interval times. The participants performed two trials (with two-minute rest intervals) on a natural grass soccer field wearing their soccer boots. Split times (5 m, 10 m, and 20 m) and total time (40 m) were recorded to the nearest 0.01 s, with the fastest time for each split used in the final analysis.

#### 2.3.3. Reactive Agility Test (RAT)

**Figure 1:**
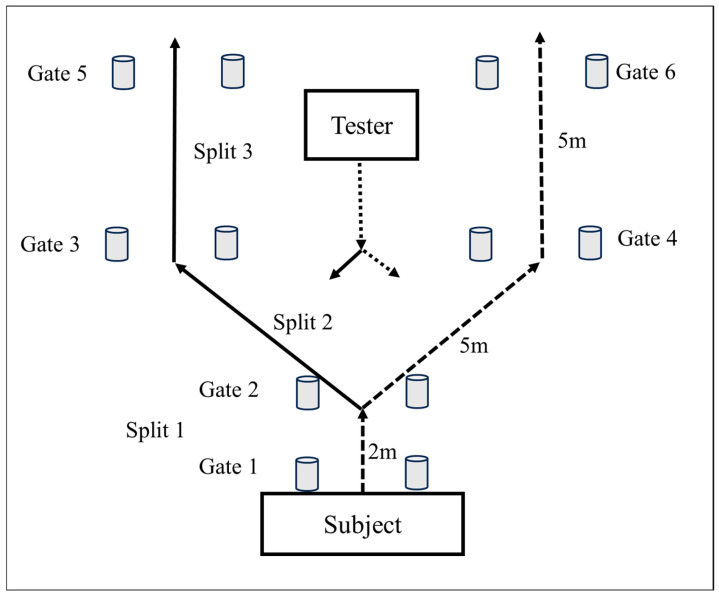
Schematic representation of the Reactive Agility Test.

The reactive agility test (RAT) [26] was set up, as illustrated in Figure 1, by using six electronic timing gates (SmartSpeed, Fusion Sport, Australia). The first gate was positioned at the start line (0 m), and the second gate was two metres straight away. Gates three and four were placed five metres to the left and right, respectively, at 45° angles, after which gates five and six were placed five metres straight in front of gates three and four. One run involved an initial forward sprint, then a pivot on the dominant foot when entering gate two, followed by a pivot on the non-dominant foot with a 45° change in direction, with a subsequent forward sprint. In contrast, the alternative option involves a pivot on the non-dominant foot, followed by a pivot towards the dominant side with a 45° change in direction. A tester (who remained the same throughout all the measurements to ensure consistency) stood 6 m in front of the starting line. Once the player initiated the sprint, the tester performed either of the following two movements in a randomised order three times:

1. Step forward to the player’s dominant side, with the player evading towards his non-dominant side.
2. Step forward to the player’s non-dominant side, with the player evading towards his dominant side.

The participants were instructed to sprint from gate one through gate two and then change direction to evade the tester (moving forward either to his dominant or non-dominant side) and sprint through either gates three and five (step to the left) or gates four and six (step to the right). The participants were directed to only respond to the change in direction, as initiated by the tester, as they would in a game situation; therefore, they moved swiftly to evade the tester.

Participants completed three trials to their dominant and non-dominant side in a randomised order within five minutes, totalling six repetitions, with the best times recorded for split 1, 2, 3, and overall time (see Figure 1). The reaction time in the agility test was calculated from the moment the athlete broke the second beam and reacted to the stimulus to the time when the athlete correctly ran through the last gate, left or right.

#### 2.3.4. Sports Vision Test (SVT)

Participants completed a sports vision test (SVT) using the Sports Vision Test (SVT) sensor pad, which features 80 touch-sensitive red-light-emitting diodes (LEDs). The SVT™ was programmed to use a proactive mode (Sport Vision, 2012), meaning that lights remained illuminated until the participant responded by hitting them. Participants stood face forward in front of the sensor pad, which randomly illuminated a sequence of 20 lights (centred on 16 lights, arranged in a 4 by 4 array). Participants were asked to hit the illuminating light as fast as possible. The total time to hit the sequence of 20 lights was recorded in milliseconds. The test began with two practice trials (for familiarisation purposes), followed by four test trials, each separated by a 10 s break. The SVT™ links to a laptop that controls the SVT™ board using a Windows-based software programme (SVT Professional Software V.1.04). Using SVT to assess hand–eye coordination has been reported as a reliable measure, with an intraclass correlation coefficient ranging from 0.74 to 0.86 across four trials [21].

### 2.4. Statistical Analysis

SPSS software (version 29, IBM) and JASP (version 0.18.3) were used to perform statistical analysis of the data gathered. Descriptive statistics (averages, standard deviation, minimum, and maximum values) were calculated for each variable, and the Shapiro–Wilk test confirmed the normality of the data distribution (*p* > 0.05). For the RAT exclusively, a paired sample *t*-test was performed to determine whether changes were present for dominant vs. non-dominant limbs. A one-way analysis of variance (ANOVA) was conducted, followed by a Bonferroni post hoc test for positional discrimination. Spearman’s rho was performed to determine relationship strengths between the test variables (speed [5, 10, 20, and 40 m distances], RAT [split 1, 2, 3, and total time], and SVT [minimum, maximum, average, and total time]). The correlation strength was interpreted as: r = <0.1, trivial; 0.1–0.3, small; 0.31–0.49, moderate; 0.5–0.69, large; 0.7–0.89, very large; and 0.9–1, perfect correlation [27]. Fisher’s Z was used to measure the effect size (ES) and interpreted as follows: trivial: <0.20; small: 0.20–0.49; medium: 0.50–0.79; large: 0.80–1.19; very large (1.20–2.0); and extremely large (>2.0) [28]. Finally, a mediation analysis was conducted to evaluate, through regression analysis, whether the mediator (e.g., SVT results) could predict (*p* < 0.05) the performance of the dependent variable (speed and RAT results) to a greater extent than in the absence of the mediator [29].

## 3. Results

Provided in Table 1 are the descriptive statistics of the physical test results. All test results for the various playing positions are provided in Table 2.

When discriminating between playing positions, the only significant difference was observed between midfielders and forwards for minimum time spent completing the SVT test (F = 3.438, *p* = 0.027).

**Figure 2:**
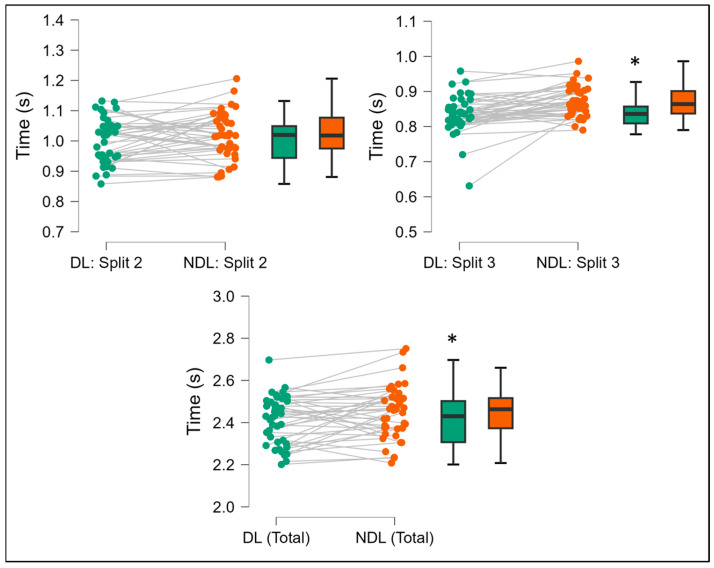
Comparison of directional changes across split times of the reactive agility test for the dominant vs. non-dominant limbs. DL = dominant limb; NDL = non-dominant limb; s = seconds; * *p* < 0.05.

As observed from Figure 2, the dominant and non-dominant limb RAT results revealed a significant difference for the total time (*p* = 0.02; ES = 0.37) and split 3 times (*p* < 0.001; ES = 0.69).

**Figure 3:**
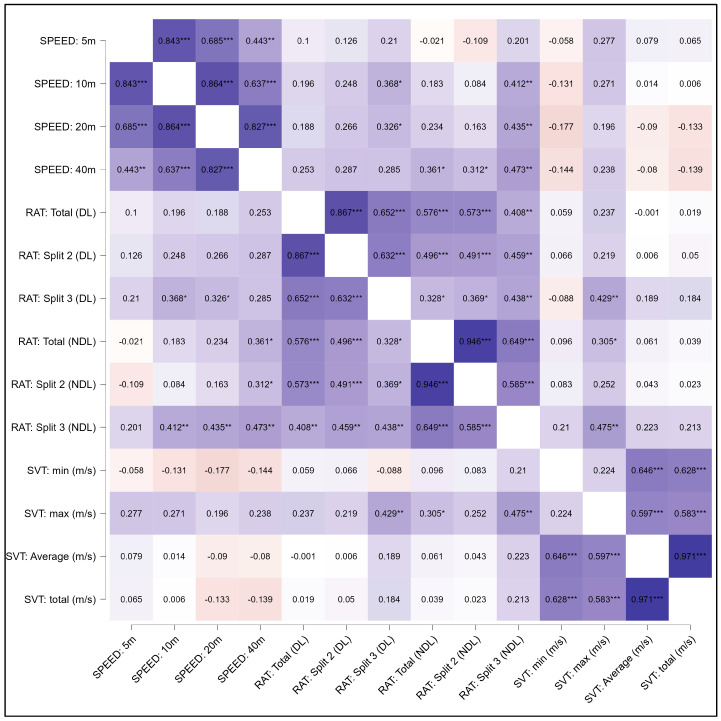
Heatmap representing correlation strength and statistical significance among speed, visual reactive, and live reactive performance metrics. * = *p* < 0.05, ** = *p* < 0.01, *** = *p* < 0.001, DL = dominant limb; NDL = non-dominant limb; SVT = Sports Vision Test; RAT = Reactive Agility Test; s = seconds; m/s = millisecond.

The levels of correlation and significance among the test variables following Spearman’s rho are illustrated in Figure 3. A small positive relationship was noted between playing experience and RAT (total—DL) (r = 0.33, *p* = 0.03, ES = 0.16) and SVT (maximum times) (r = 0.32, *p* = 0.04, ES = 0.33). As expected, significant relationships were observed between the variables within the same tests (i.e., speed, SVT, and reactive agility), while limited test relationships were observed. No significant relationships were seen between the sprint test and SVT variables. A moderate positive relationship was observed between split 3 (RAT) and the maximum SVT times (DL: r = 0.43, *p* = 0.004, ES = 0.5, NDL: r = 0.48, *p* = 0.001, ES = 0.5). In addition, a small yet positive relationship was observed between the total time completed for the RAT and the NDL, as well as the maximum SVT results (r = 0.31, *p* = 0.04, ES = 0.3).

The times recorded for RAT split 2 and 3 of the DL showed a moderate correlation with the speed distances recorded at 10 m (Split 2: r = 0.311, *p* = 0.04, ES = 0.3, Split 3: r = 0.39, *p* = 0.01, ES = 0.41), 20 m (Split 2: r = 0.34, *p* = 0.02, ES = 0.36, Split 3: r = 0.35, *p* = 0.02, ES = 0.37) and 40 m (Split 2: r = 0.36, *p* = 0.02, ES = 0.38, Split 3: r = 0.32, *p* = 0.04, ES = 0.33). Rendering the RAT results for the NDL, only split 3 correlated moderately with 10 m (r = 0.41, *p* = 0.007, ES = 0.42) and 20 m (r = 0.44, *p* = 0.005, ES = 0.44) and 40 m (r = 0.47, *p* = 0.002, ES = 0.49), with the total times obtained for the RAT to the NDL correlating with the 40 m (r = 0.36, *p* = 0.03, ES = 0.39) sprint results. Upon completing a linear regression model to investigate whether any performance measure can predict reactive agility abilities, a small but significant value was noted between the 40 m speed and overall reactive agility performances of both dominant and non-dominant limbs (*p* = 0.03, R2 = 10.5%).

## 4. Discussion

Our study examined the relationship between laboratory-based (SVT) and field-based (RAT) psychomotor reactive agility and speed tests in soccer players. Moderate correlations were found between RAT and various sprint distances, but no meaningful relationship emerged between SVT and RAT components. Additionally, position-specific RAT and SVT results did not reveal positional differences. A limited predictive relationship of 10.5% was observed between sprint acceleration and total RA times.

Soccer involves various elements, such as the ball, goal box, and players, which, when combined with the diverse actions of teammates and opponents, place significant demands on the visual system, necessitating efficient perceptual and cognitive functioning [30,31]. The speed of reaction to visual stimuli is crucial for effectively executing actions on the field [30]. Vision board training has emerged as a novel approach to improving athletes’ visual skills and decision-making abilities [32]. Vision boards typically consist of visual stimuli, such as flashing lights, moving objects, or target patterns, presented in controlled environments to simulate game-like scenarios [32]. By engaging in repetitive tasks designed to enhance visual perception and processing speed, athletes may experience improvements in reaction times [32]. However, the ecological validity of these findings—i.e., their relevance to real-life sporting contexts—remains a subject of inquiry. Factors such as stimulus specificity, task complexity, and environmental unpredictability may influence the transferability of psychomotor test results to sports performance.

A moderate correlation between the SVT and the final 5 m split of the RAT was observed. This finding is unexpected, given that the final segment of the RAT is predominantly neuromuscular and places minimal demands on athletes’ perceptual or cognitive processing. Possibly, players with faster decision-making processes can execute the COD movement sooner or more effectively. This may lead to an increase in the time they spend in a linear sprint during the RAT, resulting in higher speed production in the final 5 m split of the RAT. Vision board reactive tests engage cognitive processes such as attention, perception, and decision-making, which are fundamental to reactive performance in sports [32] and can be transferred to real-life sport scenarios [21,33]. While previous research suggests that cognitive processes may contribute to reaction performance [34], our findings indicated that light-based tests may not reflect the cognitive demands of real-game situations. Research suggests that improvements in specific perceptual–cognitive skills acquired through training can generalise to various contexts, enhancing athletes’ performance across different tasks, potentially explaining split 3 results [35].

No significant correlations were found between the RAT split 2, which requires a reaction to visual stimuli, and the SVT. This result may be attributed to the low specificity of the SVT assessment, suggesting that the reaction to light in a laboratory setting versus the reaction to a live tester in the field may rely on different cognitive and perceptual functions. The authors propose that the contrast in movement demands—SVT being a static, two-dimensional test while RAT split 2 requires dynamic, whole-body movement—could further explain the lack of significant correlations. Previous research concurred that reaction time correlates differently depending on the physical and movement demands of a task [36]. Zemková and Hamar [36] reported how reaction time correlated with reactive agility performance, but only for short repetitive distances (0.8 m; r = 0.766) and not for longer distances (1.6 and 3.2 m). This suggests that minimising motor performance contributions during RAT allows for a greater focus on perceptual–cognitive functioning in task execution. Previous findings proved that RATs can effectively detect differences between soccer players competing at different levels [15]. For example, RAT time for U19 players was shorter compared to their U17 and U15 counterparts [15]. Notably, another study identified significant differences in reaction times between elite and sub-elite soccer players using the same RAT as the current study, with no differences observed when light stimuli replaced the live tester [14]. Using the same protocol, elite adult players outperformed sub-elites during live but not during the light RATs [14]. Despite these findings, vision training remains a potentially useful approach for improving perceptual-cognitive skills. However, its effectiveness may be limited to specific domains.

Vision board tests and training require athletes to maintain attentional focus and situational awareness, skills deemed crucial for reacting effectively to dynamic game situations [37]. Studies have demonstrated that attentional control and situational awareness significantly contribute to athletes’ performance in sports that require rapid reactions [38]. In addition, vision board training provides immediate feedback on performance, allowing athletes to adjust their strategies and refine their reactive abilities [39]. Feedback supports skill acquisition [40], though its role in bridging the gap between lab-based reaction tests and on-field agility requires further investigation. While vision board tests offer standardised measures of reactive abilities, individual differences in perceptual–cognitive skills and motor proficiency exist [34]. However, studies have shown that vision board training can improve reactive performance across diverse athlete populations, with variations in initial skill levels [35].

The ability to process visual information rapidly and accurately is crucial for reacting swiftly in soccer. Athletes with faster linear speed often demonstrate superior perceptual-cognitive processing, enabling them to identify relevant cues and make rapid decisions under pressure [35]. Recently, Matlák and co [41] reported a significant correlation between choice reaction time and decision time among elite youth soccer players in the live RAT (r = 0.458). They further claimed that live reactive agility performance cannot be explained by the linear sprint speed of soccer players, which contrasts with the current findings, demonstrating that the final split of the RAT correlated significantly with acceleration (10 and 20 m) and top speed (40 m). The authors suggest that the correlations observed only for the final split of the RAT can be attributed to the contextual similarities with the 40 m sprint test. As mentioned earlier, the first two splits of the RAT demand a cognitive (i.e., visual stimuli and decision-making) and physical load (i.e., COD and sprint), while the final split of the RAT predominantly requires physical effort similar to the 40 m sprint. Hence, the absence of cognitive aspects emphasises the reliance on physical speed when performing the 40 m sprint and the final split of the RAT. This could explain the moderate relationship observed between the 20 m sprint and split 2 (RAT). However, in split 2 (RAT), effective psychomotor functioning may be essential, as both physical (speed) and cognitive (visual response) characteristics play a role. These attributes contribute to efficient movement execution, enabling players to respond rapidly to stimuli [42]. This heightened anticipation enables players to react more quickly to unfolding situations, such as intercepting passes or closing down opponents [43]. Players with a faster linear speed exhibit enhanced neural efficiency, facilitating quicker processing and execution of motor commands, which can translate into faster reaction times during soccer-specific tasks [34].

Despite the novelty of this study, the results should be interpreted with certain limitations in mind. While the light reaction test (SVT) is less biassed than the live RAT, it sacrifices ecological validity. The SVT is conducted in a stationary position within the frontal plane, limiting its ability to represent the complexity of match environments (i.e., peripheral vision, cue utilisation, and decision-making). This constrains its assessment of responsive agility, whereas an integrated approach combining cognitive and physical skills is essential for football performance. Future research should enhance the ecological validity of laboratory tests by incorporating sport-specific stimuli, such as wearable technologies (i.e., Inertial Movement Units), virtual reality, and video-based or large-screen reaction tests (see review [44]). The sample size may be insufficient to draw definitive conclusions, especially given the high individual variability typically associated with agility performance. Future research involving a larger sample size is recommended to confirm these findings. Another limitation is the lack of counterbalancing in the SVT and RAT order, which could have introduced order effects such as fatigue or learning bias. However, the study aimed to mitigate this by allowing a six-hour recovery period between tests. Implementing the recommendations presented will enhance the measurement and utilisation of both laboratory and field-based tests, particularly in the sport domain. Further research is needed to bridge the gap between lab and field tests, differentiate players’ positions, and make the assessments more useful for coaches and practitioners.

In summary, while both perceptual–cognitive and physical–motor skills are essential for developing real-life reactive agility in sports, research often emphasises the significant role of perceptual–cognitive abilities in facilitating rapid decision-making and effective responses to dynamic situations on the field or court [31]. However, a holistic approach that integrates training for both perceptual–cognitive and physical–motor skills is likely to yield the most robust improvements in reactive agility performance. Coaching staff should incorporate training to improve linear speed, as this involves motor learning and skill acquisition processes that can generalise to other aspects of performance, including reaction times. As athletes develop greater proficiency in sprinting mechanics and movement patterns, they may also exhibit improvements in their ability to react quickly to stimuli during soccer gameplay [33].

## 5. Conclusions

The motor response to human visual stimuli is a unique skill that differs from the reaction to light-based visual stimuli. The weak relationship observed between laboratory light-based reaction speed and live reactive agility performance in collegiate soccer players confirmed this notion. Reactive agility comprises three distinct phases: perception, decision-making, and execution. It was during the execution phase that acceleration speed demonstrated a modest predictive relationship with overall live reactive agility performance, which can contribute to the physical implementation of the chosen response. Lastly, live reactive agility and light-based reaction speed testing are not sensitive enough to detect positional differences among collegiate soccer players.

### Practical Applications

Practitioners should develop and implement more ecologically valid assessment tools that can be used for talent identification, athlete monitoring, and training intervention purposes tailored to the dynamic demands of team sports. A light-based reaction speed and hand-eye coordination test alone has limited ecological validity for assessing responsive agility in sport-specific scenarios. We propose the integration of a training and assessment protocol that concurrently targets cognitive and physical performance, specifically incorporating players’ reactive responses to human movement stimuli (i.e., video-based reactive drills or live opponent-based drills).

## Figures and Tables

**Table 1:** Descriptive statistics ± SD of test results. DL = dominant limb; NDL = non-dominant limb; SVT = Sports Vision Test; RAT = Reactive Agility Test; s = seconds; m/s = milliseconds.

Physical Test	Mean ± SD
Speed: 5 m (s)	1.5 ± 0.05
Speed: 10 m (s)	1.79 ± 0.06
Speed 20 m (s)	3.08 ± 0.1
Speed: 40 m (s)	5.51 ± 0.2
RAT: DL: Total (s)	2.42 ± 0.11
RAT: DL: Split 2 (s)	1.01 ± 0.07
RAT: DL: Split 3 (s)	0.89 ± 0.21
RAT: NDL: Total (s)	2.45 ± 0.13
RAT: NDL: Split 2 (s)	1.02 ± 0.08
RAT: NDL: Split 3 (s)	0.92 ± 0.2
SVT: minimum (ms^−1^)	0.28 ± 0.1
SVT: maximum (ms^−1^)	0.52 ± 0.2
SVT: average (ms^−1^)	0.36 ± 0.12
SVT: total (ms^−1^)	7.11 ± 2.38

**Table 2:** Descriptive statistics ± SD of physical test results between playing positions. DL = dominant limb; NDL = non-dominant limb; SVT = Sports Vision Test; RAT = Reactive Agility Test; s = seconds; m/s = milliseconds. * *p* < 0.05 between groups.

	Defenders *n* = 16	Forwards *n* = 14	Midfielders *n* = 10	Goalkeepers *n* = 4
Speed: 5 m (s)	1.05 ± 0.04	1.04 ± 0.04	1.07 ± 0.07	1.04 ± 0.05
Speed: 10 m (s)	1.78 ± 0.06	1.77 ± 0.05	1.81 ± 0.08	1.79 ± 0.08
Speed 20 m (s)	3.09 ± 0.1	3.06 ± 0.09	3.11 ± 0.1	3.09 ± 0.17
Speed: 40 m (s)	5.54 ± 0.19	5.42 ± 0.19	5.56 ± 0.19	5.55 ± 0.32
RAT: DL: Total (s)	2.43 ± 0.12	2.42 ± 0.12	2.40 ± 0.10	2.39 ± 0.15
RAT: DL: Split 2 (s)	1.02 ± 0.08	1.00 ± 0.07	1.00 ± 0.08	0.99 ± 0.07
RAT: DL: Split 3 (s)	0.90 ± 0.21	0.88 ± 0.23	0.91 ± 0.24	0.85 ± 0.05
RAT: NDL: Total (s)	2.48 ± 0.15	2.42 ± 0.09	2.44 ± 0.12	2.51 ± 0.15
RAT: NDL: Split 2 (s)	1.03 ± 0.10	1.00 ± 0.06	1.01 ± 0.07	1.07 ± 0.08
RAT: NDL: Split 3 (s)	0.92 ± 0.21	0.91 ± 0.19	0.94 ± 0.24	0.90 ± 0.06
SVT: minimum (ms^−1^)	0.30 ± 0.03	0.33 ± 0.04 *	0.28 ± 0.14 *	0.31 ± 0.05
SVT: maximum (ms^−1^)	0.58 ± 0.10	0.54 ± 0.13	0.59 ± 0.15	0.56 ± 0.07
SVT: average (ms^−1^)	0.39 ± 0.03	0.40 ± 0.04	0.39 ± 0.05	0.39 ± 0.04
SVT: total (ms^−1^)	7.81 ± 0.56	7.90 ± 0.83	7.79 ± 1.04	7.67 ± 0.83

## Data Availability

The data presented in this study are available from the corresponding author upon reasonable request. The data are not publicly available due to institutional ownership by the affiliated tertiary institution.

## Abbreviations

The following abbreviations are used in this manuscript:

RA	Reactive Agility
RAT	Reactive Agility Test
SVT	Sports Vision Test
COD	Change of Direction
NWU	North-West University
